# Novel arrangement for an electro-Fenton reactor that does not require addition of iron, acid and a final neutralization stage. Towards the development of a cost-effective technology for the treatment of wastewater

**DOI:** 10.1016/j.chemosphere.2018.02.036

**Published:** 2018-05

**Authors:** Dennys Fernández, Irma Robles, Francisco J. Rodríguez-Valadez, Luis A. Godínez

**Affiliations:** Centro de Investigación y Desarrollo Tecnológico en Electroquímica, CIDETEQ, Pedro Escobedo, P.O. Box 064, Querétaro, 76703, Mexico

**Keywords:** Electro-Fenton, Activated carbon, Ion exchange resin, Electrochemical reactor

## Abstract

A novel arrangement for an electro-Fenton reactor aimed to treat neutral wastewater is presented. The arrangement consists on three-compartments in series, two of them packed with a cation exchange resin and one positioned between these, containing a polarized activated carbon column where the electrochemical generation of the Fenton reagent takes place. While the hydroxyl radicals electrochemically produced *in-situ*, react with the pollutant species adsorbed on the activated carbon cathode, the resin compartments administrate and collect the iron cation and the hydrated proton species in alternating flow direction cycles. The resulting process is a system that does not require acid or iron chemical addition to the process while at the same time, renders decontaminated water free of iron-dissolved species at neutral pH. The proposed electrochemical reactor arrangement is therefore the basis for the design of commercially viable electro-Fenton reactors in which the addition and subsequent removal of acid and iron chemicals is avoided; two of the currently most limiting features for the development of electro-Fenton technology for treating wastewater.

## Highlights

• A novel arrangement for an alternating electro-Fenton process is proposed.
• The reactor consists on polarized activated carbon and ion exchange resin sections.
• The system avoids acid or iron addition rendering iron-free water at neutral pH.
• The approach points to the development of competitive electro-Fenton technology.

## Graphical abstract

**Figure:**
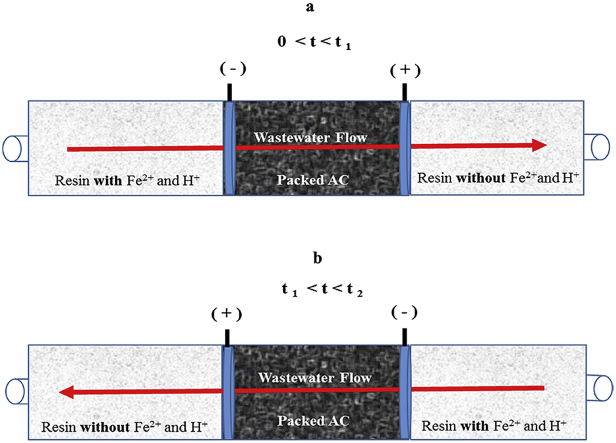

## 1 Introduction

It is well known that the availability of clean water for human consumption is one of the most urgent and important challenges that nations, all around the world, are currently facing (Luh and Bartram, 2017, Rodriguez-Narvaez et al., 2017). The need to develop efficient cost-competitive technologies to remove a wide variety of recalcitrant pollutants that cannot be dealt with using biological treatment processes, is therefore one of the main subjects of research for many groups worldwide (Alzahrani and Mohammad, 2014). In this regard, advanced oxidation processes (AOPs) constitute an attractive approach due to the high oxidation potential of hydroxyl radicals, their non-selective nature and the possibility of rendering full mineralization of a wide variety of organic pollutants in water effluents (Geissen et al., 2015). Among the various methods to produce hydroxyl radicals, the electro-Fenton approach (which relies on the electrochemical production of H_2_O_2_ via the reduction of dissolved O_2_ in the presence of Fe(II)), stands out as one of the most promising approaches to develop advanced oxidation technologies (see Eqs. (1), (2)) (Peralta-Hernández et al., 2006, Bañuelos et al., 2013, Rahim Pouran et al., 2015, García-Segura et al., 2016).

(1) $$2H^{+}+O_{2}+2e^{-}\to \;H_{2}O_{2}\;$$

(2) Fe^2+^+H_2_O_2_→HO•+HO^−^ + Fe^3+^

In spite of its potential however, electro-Fenton technologies are still seriously limited by high cost features such as the need to add and maintain appropriate ionic Fe concentrations in an acidic medium and the requirement to remove the iron species and neutralize the acid of the aqueous effluent after treatment (Brillas and Martínez-Huitle, 2009, Nidheesh and Gandhimathi, 2012, Luo et al., 2014). In this communication, we are reporting a preliminary study on a novel reactor arrangement designed to overcome these important drawbacks. It is hoped that this reactor arrangement could constitute the basis for the development of efficient and cost effective electro-Fenton water treatment processes well suited for a wide variety of polluted wastewaters (Ma et al., 2016, Roth et al., 2016, Chmayssem et al., 2017).

## 2 Materials and methods

The commercially available cation exchange resin Amberlite (CER, IR-120, Fluka) was thoroughly washed with DI water and acidified with H_2_SO_4_. In a second stage, the acidified resin was partially exchanged with either FeSO_4_·7H_2_O (99.9%, JT Baker, 0.8 M) or KOH (Macron fine chemicals, 99%) as previously published by our group (Ramírez et al., 2010, Bañuelos et al., 2015). Activated carbon (AC) on the other hand, was obtained from Clarimex, (Mexico), and cleaned by immersion for 30 min under continuous stirring using 0.02 M HCl (J.T. Baker, 38%) solution, followed by treatment for 12 h employing 5% HNO_3_ solution (J.T. Baker, 70%) (Peralta-Hernández et al., 2005, Esquivel et al., 2009). The dye chosen for this study, Orange II (4-(2-Hydroxy-1-naphthylazo) benzene sulfonic acid sodium salt, 98%) was obtained from Fluka-Analytical. All the solutions employed in this work were prepared using type I water (5 μS cm^−1^), (ASTM D1193-06, 2006).

The electro-Fenton reactor on the other hand, was constructed employing three acrylic cylindrical modules (9 cm length, 46 mL per module) in a linear arrangement, coupled together using stainless steel screws. The compartment in the center contained AC with a density of 0.44 g mL^−1^ so that electric contact between the carbon particles takes place and polarization of the AC packed electrode can be carried out. The other two compartments were loaded with an iron-acid and with a sodium-acid exchanged resin, respectively, that were prepared as previously described. The three compartments and the materials contained in each one, were separated by a carbon cloth that also worked as an electrical contact for the polarization of the AC packed electrode in the middle compartment. The polarization across the AC packed electrode in the middle compartment of the reactor (0.7 V between the two carbon cloth contacts (Bañuelos et al., 2013), as well as the associated electrochemical oxygen reduction reaction (see Eq. (1)), were promoted using a PAR potentiostat (model METEK VersaSTAT 4). The oxidation extent of the dye was monitored by the absorbance decrease (λ = 480 nm) of an Orange II solution (4.3 × 10^−4^ M) that was prepared using 0.05 M Na_2_SO_4_ as supporting electrolyte. The pH and absorbance determinations were carried out using an Orion VersaSTAT 90 Potentiometer and an Agilent Technologies UV–Vis spectrophotometer (model 8454), respectively. All experiments were carried out at room temperature with a constant flow rate in the reactor (15 mL min^−1^) that was maintained using a Cole-Palmer Masterflex 77200-62 pump.

## 3 Results and discussion

### 3.1 Conceptual design

**Fig. 1:**
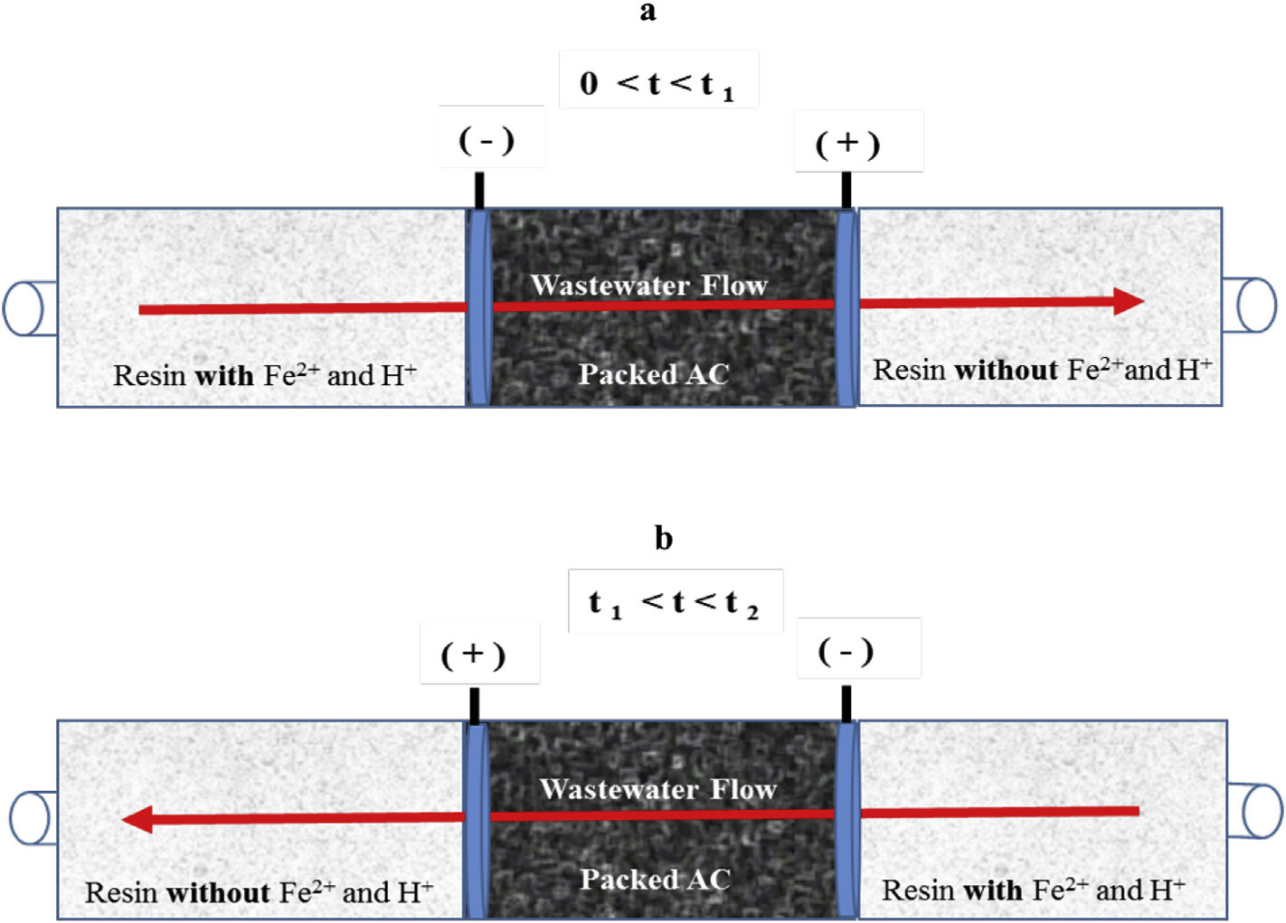
Schematic representation of the alternate polarization-flow direction performance of the proposed electro-Fenton reactor in which Fe-free, neutral wastewater is treated.

As can be seen in Fig. 1a, the reactor under study consists on the linear arrangement of three sections in which the central one consists in a polarized AC packed electrode. This section of the reactor performs two simultaneous functions. On one hand, it works as the adsorbent material for pollutants flowing across the packed AC and on the other, as the region in which the pollutant is oxidized at the AC-solution interphase either by the HO^•^ radicals produced by the electro-Fenton reaction (see Eq. (2)), or by direct electro-oxidation (Sirés et al., 2014, Pérez et al., 2017).

For the electro-Fenton reaction to occur however, it is necessary to have oxygen and Fe(II) in acidic medium at the central compartment (see Eqs. (1), (2)); a condition that is provided by the cation exchange resin in the far-left side of Fig. 1a, as an originally neutral (neutral pH conditions were chosen not only because pH around 7 is common in wastewater, but also because at this pH the system is relatively separated from the ideal acidic environment in which the electro-Fenton reaction takes place, thus providing proper experimental conditions that will allow the concept of this study to be tested), Fe-free contaminated solution, flows through this compartment and washes out the cationic Fe and H species taking them into the central compartment of the reactor. As it shown in Eqs. (1), (2), these chemicals participate in the electro-Fenton reaction in the central section of the reactor, and eventually leave this section (Probably an important fraction of ionic Fe in the form of Fe(III) due to electro-oxidation of Fe(II)) to be electrostatically retained in the far-right section of the reactor where a K loaded cation exchange resin is located. Inspection of Fig. 1a also shows that, as long as the Fe and H species are not washed out from the far-right compartment, the system would be delivering neutral electro-Fenton treated Fe-free water.

The described situation holds from the beginning of the experiment, *t* = 0, up to a time *t*_1_ in which the ionic Fe and H start to leave the reactor. After that time, at *t*_1_ ≤ *t* ≤ *t*_2_, *t*_2_ = 2*t*_1_, the AC polarization as well as the flow direction of the aqueous effluent can be reversed (see Fig. 1b) and the idealized description of the processes would be symmetric and essentially the same to that shown in Fig. 1a.

Extending this description to polarization and flow direction cycles defined by frequency 1/*t*_1_, it is possible to foresee a continuous electro-Fenton reactor that treats neutral waste-water. The arrangement can be thought as a system in which, by virtue of the location of the cation exchange resin compartments, the Fe and H reagents are held all the time within the reactor and therefore, the process does not require the addition nor the removal of Fe salts or acid, and the neutralization of the treated effluent in a subsequent stage.

### 3.2 Transport experiments

**Fig. 2:**
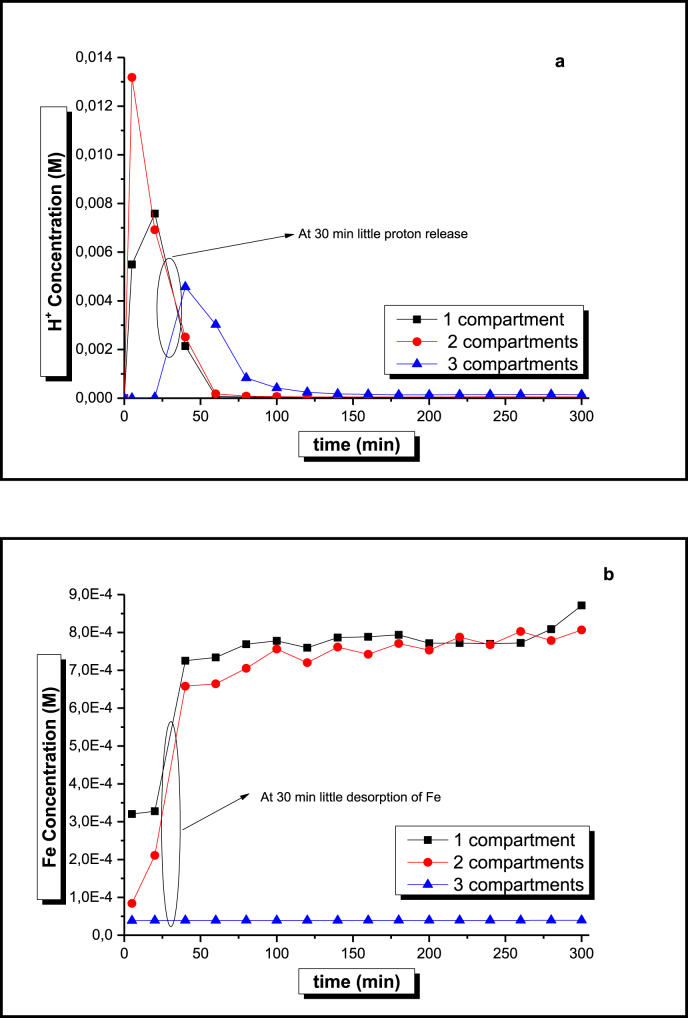
a, proton, and b, Fe transport experiments using one, two and the three compartments of the reactor schematically shown in Fig. 1. Constant flow rate of 15 mL min^−1^ at 298 K of an Fe-free, pH 7, aqueous solution.

To test these ideas, the first step is to determine *t*_1_ (and the corresponding frequency, 1/*t*_1_), by means of transport experiments for ionic Fe and H across the different sections of the reactor schematically shown in Fig. 1. In this way, experiments measuring the pH and the concentration of ionic iron were performed feeding the resin compartment, the coupled resin and AC loaded chambers and the complete three compartment arrangement shown in Fig. 1, with a neutral iron-free aqueous solution (K_2_SO_4_, I = 0.01) at a constant flow rate (see Fig. 2).

Fig. 2a shows the results of proton transport experiments. Inspection of this data, reveals that confinement in each one of the three sections under study is rather weak. In this way, while the one and two compartment systems liberate the acid contained in the resin, as well as that in the resin and the AC material (note that the acid concentration increases for the later) in about 60 min, the three-compartment arrangement, retains the acid about half an hour. After this time, H^+^ comes out from the reactor achieving a maximum concentration at 40–50 min, followed by a continuous decreasing concentration curve that reaches neutral conditions at about 180 min.

Due to charge density differences between cationic H and Fe, the results of Fe transport experiments were expected to be different to those shown in Fig. 2a. As can be seen from the corresponding data presented in Fig. 2b, while Fe ions are strongly bound by the resin, there is almost no retention by the AC material (note the similarity between the one and two compartments curves in Fig. 2b at t > 40 min). In fact, Fig. 2b shows that resin induced Fe retention is so effective that when the three-compartment reactor arrangement is employed, no Fe ions could be detected in the effluent even after 5 h.

Analysis of the data presented in Fig. 2 for the transit of ionic iron and protons across the reactor, suggests that a good approximation for *t*_1_ would be 30 min. In this way, at any time t < 30 min, that is, t < *t*_1_, it could be assumed that most of the ionic Fe and solvated protons are contained within the reactor, and that by changing the direction of the aqueous solution flow every 30 min (frequency 1/*t*_1_), most of the iron and acid can be retained within the reactor.

### 3.3 Azo-Dye degradation using the proposed Electro-Fenton reactor

Once *t*_1_ was determined, the concept described in 3.1 was tested by studying the discoloration of an aqueous Orange II solution using the three-compartment electro-Fenton reactor shown in Fig. 1. The experiments consisted in a combination of tests in which pure adsorption by the AC packed bed was examined, then, polarization was incorporated in order to produce the Fenton reagent in the middle chamber and finally flow direction and polarization were switched with a frequency 1/*t*_1_.

**Fig. 3:**
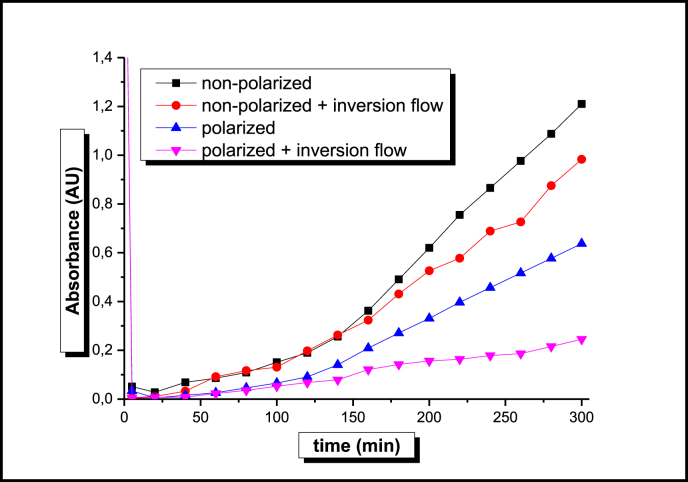
Absorbance *vs* Time data for discoloration experiments of a dye aqueous solution using alternated and non-alternated adsorption and electro-Fenton approaches.

For the simplest case in which the solution continuously flows across the reactor, adsorption of the dye on the unpolarized AC is the only absorbance reduction effect that can be anticipated. As can be seen in Fig. 3, the corresponding curve (square marks) shows that for the first 20 min, the dye is effectively retained in the AC bed since no color could be detected in the samples taken at the outlet of the reactor. After this time, a continuous absorbance increase is observed, reflecting the gradual saturation of the AC surface by dye molecules.

Flow direction change every 30 min in the former experimental set up, results in a slightly better reactor's performance at t > 140 min. As it is shown in the corresponding data in Fig. 3 (circle marks), a relatively lower absorbance increase rate at long times, that results in an absorbance difference after 300 min of about 14%, is probably due to the improved distribution of the dye molecules on the surface of the AC that results from flow direction cycling.

Polarization of the AC contained in the central chamber of the reactor, results as expected, in a substantially better performance of the reactor in terms of dye discoloration. The corresponding data in Fig. 3 (triangle marks) suggests that the polarization effect is a combination of factors such as electro-sorption, electro-oxidation and electro-Fenton discoloration promoted by electrochemically produced H_2_O_2_ in the presence of Fe and H cations (Bañuelos et al., 2013). Inspection of this data also shows that the polarized AC experimental response could be described as two linear regions of different slope that intersect at t = 120 min. According to the H transport information presented in Fig. 2a, this is the time that takes for the acid contained in the first resin loaded compartment to transit across the whole length of the reactor. The change of slope at t > 120 min, therefore suggests that from this time on, the Electro-Fenton reaction takes place in the absence of H^+^ and therefore, a smaller rate of color removal is observed.

Consistent with these observations, flow direction and polarization switching with a frequency 1/*t*_1_, results in the curve drawn with the inverted triangles in Fig. 3. Its inspection shows that the change of slope at 120 min previously described for the electro-Fenton non-switched experiments, does not take place, suggesting that as expected, the switching at time *t*_1_ intervals maintains the Fe and the acidic conditions within the reactor, thus optimizing the device performance. In this way, after 300 min of the experiment, the discoloration extent observed in the effluent is 66% larger than that obtained with the continuous electro-Fenton experiment.

## 4 Conclusions

In this way, while on one hand an activated carbon column is simultaneously employed as an adsorbent and a cathode in an electro-Fenton reactor to achieve high oxidation power at the electrode solution interphase, a couple of Fe-loaded resin compartments positioned as shown in Fig. 1, are used to avoid the need to add the Fe salts and the acid at the reactor's inlet and to remove the cations after the treatment; thus, rendering neutral decontaminated water.
